# Smartphone-Assisted Protein to Creatinine Ratio Determination on a Single Paper-Based Analytical Device

**DOI:** 10.3390/molecules26206282

**Published:** 2021-10-17

**Authors:** Izabela Lewińska, Karolina Kurdziałek, Łukasz Tymecki

**Affiliations:** Faculty of Chemistry, University of Warsaw, Pasteura 1, 02-093 Warsaw, Poland; k.kurdzialek@student.uw.edu.pl (K.K.); l.tymecki@uw.edu.pl (Ł.T.)

**Keywords:** protein, creatinine, paper-based analytical devices, smartphone, albumin to creatinine ratio, proteinuria, tetrabromophenol blue, 3,5-dinitrobenzoic acid

## Abstract

Proteinuria is a condition in which an excessive amount of protein is excreted in urine. It is, among others, an indicator of kidney disease or risk of cardiovascular disease. Rapid and reliable diagnosis and monitoring of proteinuria is of great importance for both patients and their physicians. For that reason, a paper-based sensor for proteinuria diagnosis was designed, optimized, and validated utilizing smartphone-assisted signal acquisition. In the first step, a few commonly employed protein assays were optimized and compared in terms of analytical performance on paper matrix. The tetrabromophenol blue method was selected as the one providing a sufficiently low limit of detection (39 mg·L^−1^) on the one hand and appropriate long-term stability (up to 3 months) on the other hand. The optimized assay was employed for protein-to-creatinine ratio (PCR) determination on a single paper-based sensor. For both analytes the linear ranges were within the clinically relevant range. The analytical usefulness of the developed sensors was demonstrated by a PCR recovery study in artificial urine. The obtained PCR recoveries were from ca. 80 to 150%.

## 1. Introduction

Urine of a healthy person contains only a trace amount of protein. Proteinuria is a condition in which an excessive amount of protein is excreted in urine. However, more often, the concentration of albumins is quantified in urine and this disease is referred to as albuminuria (if albumin excretion is > 300 mg/day) or microalbuminuria (if albumin excretion is < 300 mg/day) [1]. The presence of proteins in urine can be an early indicator of a kidney disease or chronic kidney disease progression. Moreover, it can be a predictor of an increased risk of cardiovascular disease or even stroke or myocardial infraction [2].

The composition of urine fluctuates during the day; therefore, protein determination in urine from 24 h collection is recommended. However, such a procedure is burdensome for the patient and the risk of inappropriate collection is relatively high [3]. Proteinuria diagnosis based on the analysis of urine from a spot collection would be much more convenient. Due to the fact that the rate of glomerular filtration is relatively stable, creatinine (excreted in urine via glomerular filtration) is commonly employed as a urine dilution marker. Correlation between albumin quantified in urine from 24 h collection and the albumin to creatinine ratio in urine from spot collection has been established and such a ratio is widely used in clinical analysis [4].

Albumin to creatinine ratio determination in urine from spot collection corresponds well with point-of-care testing principles. In fact, dipsticks for qualitative or semiquantitative urinary protein or protein to creatinine ratio determination have already been commercialized. However, they suffer from relatively low sensitivity and specificity [5]. On the other hand, in the recent years, paper has gained significant attention as a solid support for point-of-care sensors due to its low cost, low reagent consumption, disposability, lightweight, green-chemistry compatibility, and easy modification [6]. Moreover, fluids are transported on paper via capillary action, therefore no auxiliary equipment, such as pumps, is required. As a result, numerous microfluidic paper-based analytical devices (µPADs) have been reported for the determination of various analytes [7].

Some attempts to determine urinary protein with the aid of µPADs have already been undertaken. In general, two approaches can be distinguished: either determination with prior preconcentration or without preconcentration. The preconcentration process on paper has been achieved by field amplification stacking [8], isoelectric focusing [9,10], or ion concentration polarization [11]. In such systems, the limit of quantification reaches a few milligrams of protein per liter. If such a low limit of quantification value is not required, the preconcentration step can be omitted. With this respect, an origami-like 3D paper-based sensor was introduced for simultaneous determination of protein and glucose [12]. Citterio’s group reported a text-displaying paper-based analytical device for semiquantitative urinary protein determination [13]. Last but not least, a comparison between six protein assays was performed to select the one with the best analytical parameters [14].

Two groups have demonstrated paper-based analytical devices for the determination of the albumin to creatinine ratio. Chaiyo et al. used bromocresol green dye to determine the sum of albumin and creatinine concentration and picric acid for creatinine level determination. The albumin concentration was then calculated by subtraction (C_Alb_ = (C_Alb_ + C_Cre_) − C_cre_) [15]. In the other study, albumin and creatinine concentrations were determined by distance-based measurements in two separate microfluidic channels. The albumin channel had tetrabromophenol blue dye deposited in it while the creatinine channel was sensitized with Chrome azurol S and PdCl_2_ [16]. The degree of proteinuria was then semi-quantitatively assessed by drawing a line connecting the tops of the two color-changed zones.

The presented research aimed to design, develop, and optimize a paper-based sensor for protein to creatinine ratio assessment. In order to achieve this goal, two analytes had to be determined on a single µPAD simultaneously. In the first step, we optimized and compared a fewprotein assays in terms of analytical performance in paper-based sensors. Surprisingly, we found that two commonly used protein assays do not give any measurable color change when performed within the paper matrix. Owing to a careful condition optimization, the obtained analytical parameters for protein determination methods significantly exceed the ones presented in [14]. The best performing protein assay was then used for protein measurement to determine the protein to creatinine ratio while creatinine was quantified according to our previous study [17] with 3,5-dinitrobenzoic acid. Signal acquisition was accomplished with an appropriately modified smartphone to further increase the potential of the developed sensors as point-of-care tests, which makes the presented research the first smartphone-assisted PCR quantitative analysis. Moreover, to determine the protein to creatinine ratio using the obtained µPADs, no auxiliary equipment is required besides a smartphone. Finally, the possibility of calibration-free protein to creatinine estimation was evaluated.

## 2. Materials and Methods

### 2.1. Materials and Instruments

Creatinine, ponceau S (PS), bromocresol green (BCG), tetrabromophenol blue (TBPB), Coomassie Brilliant Blue G (CBB), copper (II) nitrate, and 3,5-dinitrobenzoic acid (DNBA) were purchased from Merck (Darmstadt, Germany). Bovine serum albumin, 98% purity (BSA), pyrogallol red (PR), and disodium salt of bicinchoninic acid (BCA) were obtained from Chemat (Gdansk, Poland). Other reagents of analytical grade were purchased from Avantor Performance (Gliwice, Poland). All chemicals were used without further purification. Water used in all experiments was purified with the HLP5 water purifying system (Hydrolab, Poland). BSA and creatinine standards were prepared in 0.15 mol·L^−1^ sodium chloride solution. BSA was used as a model protein standard. Whatman Qualitative Filter Paper Grade 1 and Grade 4, differing in pore size, were purchased from Merck (Germany). If needed, they were laminated with 100 µm laminating films using a typical office laminator.

### 2.2. Sensor Preparation

**Figure 1:**
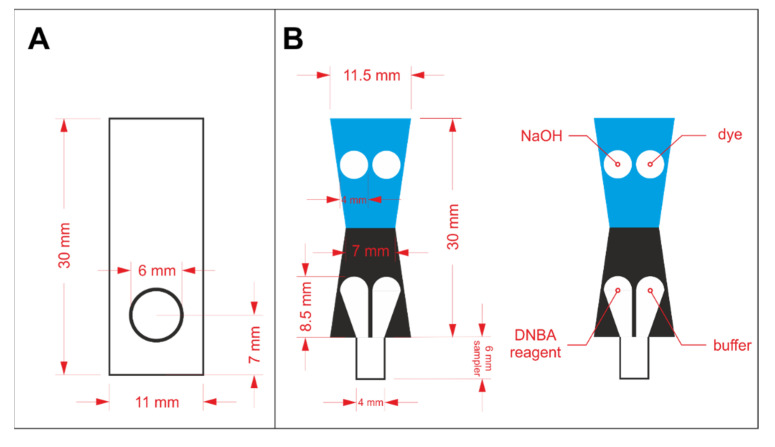
The design of wax barriers for: (**A**) protein assay optimization and (**B**) protein to creatinine ratio determination.

In order to prepare a properly operating sensor using paper as solid support, hydrophilic and hydrophobic regions must be defined. Hydrophobic barriers prevent reagents and samples from spreading in an undefined way and they are necessary for appropriate precision and reproducibility of the sensors. In this work, hydrophobic barriers were manufactured by means of the wax printing technique [18]. The hydrophobic barriers were designed in CorelDraw software and printed on Whatman paper with a wax printer (ColorQube 8570, Xerox, CT, USA). The architecture of the hydrophobic barriers for protein assay optimization and protein to creatinine ratio determination is shown in Figure 1A,B, respectively. In the latter case, the key aspect in the sensors’ design is splitting the stream of the flowing sample in a reproducible way, so that the sample can react independently with two different sets of reagents. The sensors shown in Figure 1A were printed on Grade 1 paper, while the ones shown in Figure 1B were printed on Grade 4 paper. The printed paper was heated for 2 min at 120 °C in a laboratory dryer to allow the wax to melt and penetrate the pores in the paper’s volume. After the wax solidified, hydrophobic barriers were defined.

The reagents were drop casted in the hydrophilic regions and left to dry in the ambient conditions. For the initial experiments employing the paper-based sensors shown in Figure 1A, 2 µL of reagent containing protein-binding dye was pipetted on the circular zone. Reagents required for both protein and creatinine determination were drop casted in appropriate zones, according to the scheme shown in Figure 1B. The volume of each reagent was reduced to 1.5 µL to prevent reagents from escaping their designated zones. In each case, the deposition of reagents was repeated twice. Paper-based devices for protein assays’ comparison were laminated from one side to increase rigidity. Additionally, lamination allowed less grainy photos of the detection zone to be obtained (otherwise, the roughness of paper could be seen in the photos). µPADs for protein to creatinine ratio determination were cut out and folded following the line between the blue and black region (see Figure 1B) prior to lamination, similarly to our earlier work [17]. Then, they were placed between two laminating films and passed through an office laminator. The details of these sensor preparation steps are shown in Figure S1 in the Electronic Supplementary Information (ESI).

### 2.3. Sensing Procedure

For protein assays’ optimization and comparison, 2 µL of BSA standard was introduced into the detection zone with previously deposited reagents. After an appropriate time (ca. 15 min), the sensor was placed in the dedicated smartphone case (described in more detail in Section 2.4) and a photo was taken.

In the case of sensors for protein to creatinine ratio determination, prior to sample introduction, the bottom part of the sensor’s sampler was cut off. Then, the bottom part of the sampler was dipped in the sample and held in such a position for a strictly defined time, during which the sample filled the entire hydrophilic zone, owing to capillary forces. After that, the entire sampler was cut off to eliminate the possibility of transferring the sample to the smartphone case. The sensors were incubated for a certain period of time to allow the color to fully develop. Then, they were placed in the smartphone case and a photo was taken. For more detail on the analytical procedure, refer to [17] and Figure S1 (steps E–H). Unless stated otherwise, each measurement was conducted in triplicate.

### 2.4. Smartphone Modification and Signal Processing

A smartphone, Samsung Galaxy A5 (Samsung Electronics, Suwon, South Korea) with 13 MPx camera, was used to acquire photos of the colorful detection zones in paper-based sensors. The smartphone was modified with 3-D-printed elements to ensure repeatable placement of the sensor as well as to limit the influence of outside lightning conditions on the obtained results. Such details of smartphone modification have already been shown elsewhere [17]. Briefly, the sensors were illuminated from behind with the smartphone’s flashlight, guided by aluminum foil. A macro lens was placed on the smartphone’s camera to allow for sharp photos to be taken from a close distance (2.2 cm). Between the sensor and the lens, a circular element with aperture was mounted to ensure that only the area of interest was in the taken photo. For protein to creatinine ratio measurements, this element was switched for a similar one but with two apertures, because two detection zones were present in the paper-based sensor. The photos were captured with the OpenCamera v 1.45.2 application (by Mark Harman, available in Google Play), because, contrary to the build-in-one, it allows for selection of the ISO parameter and white balance. White balance was fixed at the “warm” setting and the ISO parameter was separately optimized for each assay.

The taken photos were transferred to a PC and analyzed with ImageJ software (National Institutes of Health, Bethesda, MD USA). In each photo, a circular region-of-interest (ROI) was selected in the middle of the detection zone. Then, using the RGB Measure function, average R, G, and B intensities from the RGB color space were measured in the area within ROI. R, G, and B are within the range from 0 to 255 in an 8-bit RGB model. For each assay, the channel providing the highest sensitivity was selected and treated as analytical signal.

### 2.5. Artificial Urine Preparation

Artificial urine was prepared according to recipe [19] with minor modifications. Therefore, it consisted of 170 mmol·L^−1^ urea, 90 mmol·L^−1^ sodium chloride, 25 mmol·L^−1^ ammonium chloride, 25 mmol·L^−1^ sodium carbonate, 10 mmol·L^−1^ sodium sulfate, 7 mmol·L^−1^ potassium dihydrogen phosphate, 7 mmol·L^−1^ dipotassium hydrogen phosphate, 2.5 mmol·L^−1^ calcium chloride, 2 mmol·L^−1^ magnesium sulfate, 2 mmol·L^−1^ citric acid, 1.1 mmol·L^−1^ lactic acid, and 0.4 mmol·L^−1^ uric acid dissolved in distilled water. Artificial urine was then titrated to pH 6 with 1 mol·L^−1^ hydrochloric acid. Appropriate amounts of BSA and creatinine were dissolved in artificial urine solution and diluted with the same solution for the recovery study of the protein to creatinine ratio.

## 3. Results and Discussion

### 3.1. Dye-Binding Assay Optimization

Six commonly used protein assays were selected for optimization and analytical performance comparison in paper-based analytical devices. The principles of these assays and the initial chemical conditions are summarized in Table 1.

Surprisingly, neither Bradford nor BCA-Cu(II) assays generated any measurable color change when transferred from conventional spectrophotometry to paper-based sensor. Pokhrel et al. [14] recommended performing the Bradford assay on glass microfiber instead of paper. However, we found this remedy to be insufficient to solve the problem of a lack of color change. When it comes to the BCA-Cu(II) protein determination method, the limit of quantification for this assay performed on paper was reported to be 1200 mg·L^−1^ [14], which is above the range of interest for urinary protein determination. Moreover, in the cited work, the sample was introduced first and the reagent was added afterwards, which makes this assay unsuitable for on-site sensing. Additionally, in comparison with other tested protein assays, the reaction between BCA-Cu(II) and protein proceeds relatively slowly (at least 30 min incubation time in an elevated temperature [24]). As a result, water evaporates from the paper matrix before the colorful complex is formed in an amount allowing for sensitive detection. Obviously, the reaction cannot proceed if all the substrates are present in a dry form. Considering the above mentioned, further experiments concerning Bradford and BCA-Cu(II) assays were abandoned.

The Ponceau S method is regarded as very selective and is often employed in clinical laboratories [25]. However, the multistep procedure, which includes, among others, centrifuging, did not allow for a successful assay’s transfer to paper. On the other hand, this method is widely employed for staining protein after chromatography or electrophoresis, and it was reported to work on nitrocellulose as well [26]. We also found that when the protein sample is spotted on the paper first and then PS-TCA reagent is introduced, a measurable color change can be registered after the background destaining procedure. This approach is, again, unsuitable for sensors operating according to point-of-care principles; therefore, the PS assay was not subjected to further optimization.

Bromocresol green, pyrogallol red, and tetrabromophenol blue assays were successfully conducted using µPADs; therefore, they were chosen for further experiments. The optimization was executed using the one-variable-at-a-time approach. The variables selected to be optimized were the dye concentration, buffer pH, buffer concentration, reaction time, and ISO parameter of the smartphone’s camera (which refers to camera’s sensitivity to light). The results of the optimization experiments are summarized in Table 2 while the initial conditions are shown in Table 1.

In the beginning, it was necessary to select the channel from the RGB color space, which would be treated as the analytical signal (red, green, blue, or their mathematical transformations). The choice of the optimal channel depends on the spectral characteristics of the product being detected. For the BCG and TBPB assays, the red channel was selected as the analytical signal due to the fact that it provides the most sensitive response. The calibration curves for all three channels for the BCG method and TBPB method are shown in Figure S2 A and B in ESI, respectively. The response of the PG-based sensors in the RGB color space is somewhat different. As depicted in Figure S2 C, a decrease of R channel intensity and a simultaneous increase of B channel intensity is observed with an increasing protein concentration. This means that both of the mentioned channels can be employed as analytical signals. However, neither the precision nor sensitivity are satisfactory for accurate protein determination. For this reason, the blue to red channel ratio was calculated and treated as the analytical signal for the pyrogallol red assay. Such a procedure resulted in at least three improvements in analytical performance: (i) improved sensitivity; (ii) improved precision; and (iii) minimization of the inconsistencies in lightning conditions or white balance between measurements (they would be reduced when the ratio is calculated).

The optimization experiments led to a list of various conclusions. Those especially worth highlighting are as follows: firstly, in all cases, the optimal buffer pH is higher for paper-based sensors than for standard procedures reported in the literature. This is probably due to the fact that paper has slightly acidic pH–around 6. We performed additional experiments to estimate the pH of Whatman filter paper–phenol red indicator (transition from yellow to pink at pH 6.8–8.2) turned yellow when spotted on paper while bromothymol blue indicator (transition from yellow to blue at pH 6.0–7.6) appeared green. The results of this study justify the assumption that the pH of Whatman filter paper is in the range from 6.0 to 6.8, which means that assays requiring acidic conditions need slightly higher pH of the buffer solution when performed on paper in comparison to standard bulk methods.

In the case of the PR method, the 1 mmol·L^−1^ dye concentration gave the best results: the biggest sensitivity and the widest linear range. However, the solution was highly unstable, which lead to low reproducibility. For this reason, 0.5 mmol·L^−1^ pyrogallol red was selected as the optimal concentration, providing similar sensitivity but a narrower linear range. Last but not least, it is important to stress that for BCG and PR assays, the appropriate dye was dissolved in the buffer solution, whereas for TBPB assay, dye solution and buffer solution were introduced into the detection zone separately. This is due to the fact that in the latter case, the sensitivity was improved in comparison to using dye dissolved in the buffer solution.

Finally, for all three kinds of sensors, the dependencies of the analytical signal on time elapsed from sample introduction were registered for three concentrations of BSA: 100, 300, and 750 mg·L^−1^. The obtained kinetics are shown in Figure S3 in ESI (for clarity, only results for 300 mg·L^−1^ are plotted). Based on these experiments, the optimal reaction time between the deposited reagents and the protein-containing sample was established. For BCG-based sensors, the time from sample introduction to capturing a photo of the detection zone should be at least 15 min whereas for PG- and TBPB-based sensors, it should be at least 12 min.

### 3.2. Assay Comparison

**Figure 2:**
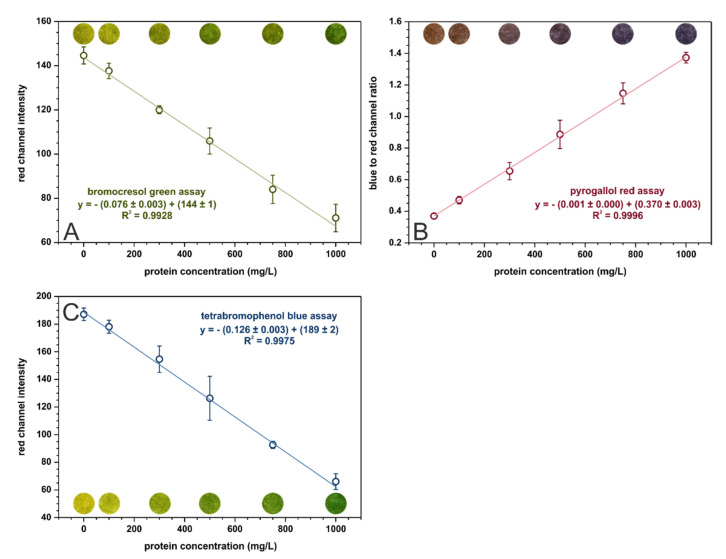
Calibration dependencies for (**A**) bromocresol green, (**B**) pyrogallol red, and (**C**) tetrabromophenol blue assays registered in the optimal conditions, *n* = 3. The colorful spots are the actual photos of the detection zones of paper-based sensors after the introduction of sample with an appropriate BSA concentration.

In the optimized conditions, calibration curves were registered for each assay in the range from 0 to 1000 mg·L^−1^ of protein. These curves, with fitted linear regressions, are shown in Figure 2. The analytical parameters calculated for three kinds of paper-based sensors are given in Table 3. The tetrabromophenol blue assay is superior in terms of analytical performance in µPADs to the bromocresol green assay. It offers better sensitivity and precision as well as lower limits of detection and quantification. The sensitivities of the BCG and TBPB methods cannot be directly compared with the sensitivity of PR-based sensors since the parameters on the *Y*-axis are different. Nonetheless, the pyrogallol red assay provides the lowest limits of detection and quantification out of the examined methods. On the other hand, the bromocresol green method is the only one that allows for selective albumins’ determination while the other methods determine all proteins.

Our findings confirm the results presented in [14], which indicated that the tetrabromophenol blue method provides the best analytical parameters for protein determination on paper (the pyrogallol red method was not included in the referenced study). However, the limit of quantification obtained in [14] for the TBPB assay equal to 2900 mg·L^−1^ does not allow for urinary protein determination in a clinically relevant range. This almost 25-fold decrease of LOQ obtained in the presented research (see Table 3) is probably attributed to firstly, using a smartphone adapter to separate the sensor from ambient lightning, and secondly, to taking into account a larger number of parameters affecting sensitivity when performing optimization (for example, buffer pH and concentration).

**Figure 3:**
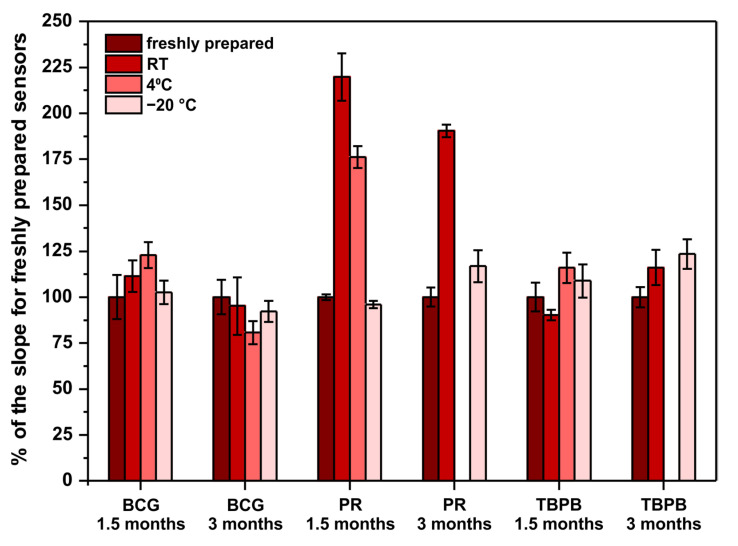
Sensors’ long-term stability study. The parameter on ordinate is the percent of the obtained calibration curve’s slope in comparison to the slope for freshly prepared sensors—100% would mean that the sensors do not change their sensitivity when subjected to storage. Note that there are no bars for PR and TBPB stored in 4 °C for 3 months due to sensors’ destruction by moisture in the fridge.

Another important factor to be considered when developing point-of-care sensors is their long-term stability. This is because such sensors should be characterized with an appropriately long shelf-life. Ideally, they should withstand storage in room temperature as there might be limited access to cooling devices at the places of sensors’ use. For this reason, the long-term stability of the developed paper-based analytical devices was examined. Sensors operating according to previously optimized methods were stored in room temperature, 4 °C (fridge), and −20 °C (freezer) for 1.5 and 3 months. After the mentioned periods of storage, the performance of the stored sensors was compared with freshly prepared sensors. Freshly prepared sensors were made on the day of measurements with reagents prepared on that day from the solvents and chemicals listed in Section 2.1. The results obtained in this experiment are shown in Figure 3.

First of all, PR-based sensors definitely require −20 °C to withstand long storage, which limits their application as point-of-care tests. Surprisingly, instead of an expected decrease of sensitivity, the obtained results clearly indicate a significant sensitivity increase. To the authors’ best knowledge, there are no reports in the literature about the stability of PR-molybdate complex and its influence on the method’s sensitivity towards protein. Quite the opposite, the literature reports that the reagents (PR, molybdate, and buffer separately) are stable when stored in room temperature, protected from light, for up to 6 months [21]. This suggests that a reaction occurs between some of the reagents (PR, molybdate, succinate, oxalate, benzoate), resulting in an increase of sensitivity towards protein. This phenomenon might be interesting to study further to increase the sensitivity of the PR assay in a controlled manner. Secondly, the fact that PR- and TBPB-based paper sensors were damaged due to moisture present in the fridge after 3 months of storage indicates that low-humidity conditions are required for long-term storage. Last but not least, both BCG- and TBPB-based sensors can be stored even in room temperature for up to 3 months without a significant change of their sensitivity, which makes them good candidates for point-of-care tests.

To conclude, despite the fact that the pyrogallol red method offers the best analytical parameters, the developed PR-based sensors cannot be stored unless a low temperature is provided. For this reason, the tetrabromophenol blue method should be selected as the optimal one to determine proteins on paper. The linear range of this assay is within the clinically relevant range of protein, the precision is satisfactory for such a simple sensing device, and the developed µPADs can be successfully stored in room temperature for up to 3 months.

### 3.3. Protein to Creatinine Ratio Determination

In the next step of the project, we further increased the usefulness of the developed sensors as point-of-care tests by including the creatinine determination zone for protein to creatinine assessment. The architecture of the paper-based analytical device was changed to the one shown in Figure 1B. For the determination of two different analytes, namely protein and creatinine, on a single paper strip, it was necessary to search for conditions allowing for sufficiently sensitive determination of both compounds. Creatinine was determined according to a previously established protocol [17]: 0.3 mol·L^−1^ 3,5-dinitrobenzoic acid in 0.45 mol·L^−1^ sodium hydroxide was deposited in the sampling channel and 2 mol·L^−1^ sodium hydroxide was pipetted on the detection zone. In a previous study, we established that this placement of reagents allows for uniform color development in the detection zone. The optimal reaction time between creatinine and the deposited reagents was 6 min. During that time, a purple product was formed and a linear response in the green channel from the RGB color space was obtained. The previously selected protein assay, utilizing tetrabromophenol blue as protein-binding dye, was employed for protein determination. The solvent for the dye was switched from pure ethanol to water-ethanol mixture in a 2:1 volumetric ratio. This was necessary to increase the hydrophilicity of the solution. Due to limited solubility of TBPB in water-ethanol mixture, its concentration was reduced to 5 mmol·L^−1^. The buffer solution remained the same as in the previous experiments: 250 mmol·L^−1^ citrate buffer, pH 4. TBPB reagent was introduced into the detection zone while buffer solution was drop casted on the sampling channel, according to Figure 1B.

In the first stage, the optimal sensor dipping time and incubation time was established. It is important to highlight that the required incubation time for protein determination is twice the time needed for creatinine reaction with DNBA. Moreover, the product of the latter reaction decomposes over time, leading to a decrease in sensitivity. To determine the optimal incubation time, reaction kinetics were registered for high (1000 mg·L^−1^ BSA, 10 mmol·L^−1^ creatinine) and low (200 mg·L^−1^ BSA, 2 mmol·L^−1^ creatinine) concentrations of analytes. Additionally, to investigate the influence of the sensors’ dipping time in the sample, three various scenarios were tested: dipping for 2, 5, or 8 min. The obtained kinetics are shown in Figure S4 in ESI. First of all, a. dipping time equal 2 min resulted in very low precision of the creatinine determination on both concentration levels. Therefore, it was regarded as unsuitable for further experiments. Sensor dipping time of 5 min allowed an improvement of the sensitivity in comparison to8 min dipping time for protein determination and did not significantly affect creatinine determination. For this reason, 5 min was chosen as the optimal sensor dipping time.

**Figure 4:**
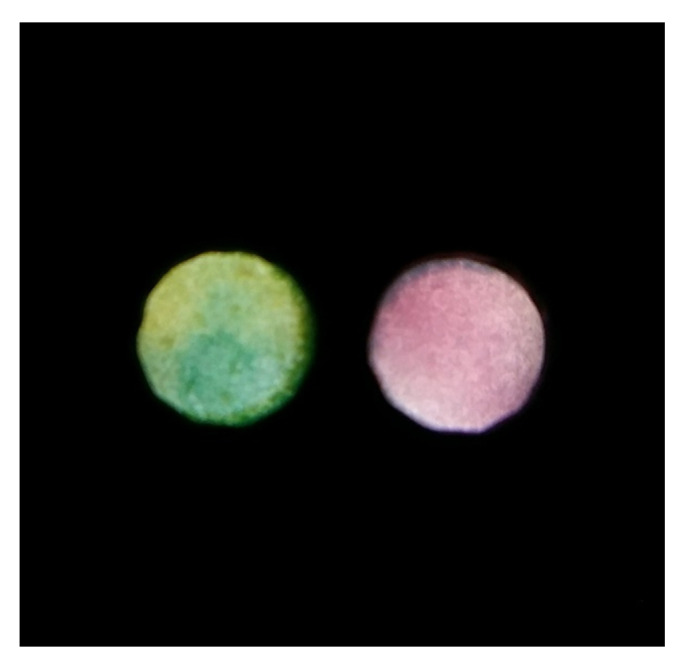
An exemplary photo obtained for simultaneous protein (left detection zone) and creatinine (right detection zone) determination.

The product of the creatinine reaction with DNBA visibly decomposed after 10 min of sample introduction, which can be seen in Figure S4 B as an increase in green channel intensity. On the other hand, the longer the reaction between protein and TBPB lasts, the better sensitivity can be achieved. To compromise these two findings, a 10 min incubation time was selected as the optimal one. Note that it means that the sensors were dipped in the sample for 5 min, removed from the sample, and left to react for another 5 min. After this time elapsed, a photo of the detection zones was captured (both detection zones were captured simultaneously). An exemplary photo taken in the process of simultaneous protein and creatinine determination is presented in Figure 4.

**Figure 5:**
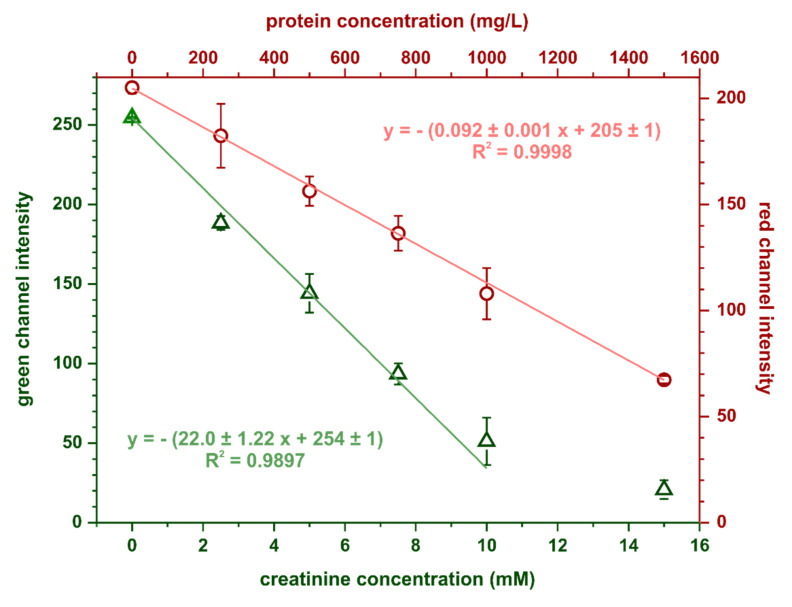
Calibration dependencies obtained in the optimal conditions for simultaneous protein and creatinine determination, *n* = 3.

The calibration curves for creatinine and protein obtained in the optimal conditions are shown in Figure 5. Limits of detection and quantification were estimated based on the following equations: LOD = 3.3SD_y_/S and LOQ = 10SD_y_/S, where SD_y_ is the standard deviation of the calibration curve’s intercept and S is its slope. For protein determination, the calculated limits of detection and quantification were 35 and 107 mg·L^−1^, respectively, while for creatinine determination, these parameters were 0.15 mmol·L^−1^ and 0.46 mmol·L^−1^, respectively. Precision, expressed as relative standard deviation, was 6% (at 750 mg·L^−1^ BSA) for the protein assay and 7% (at 7 mmol·L^−1^ creatinine) for the creatinine assay.

**Figure 6:**
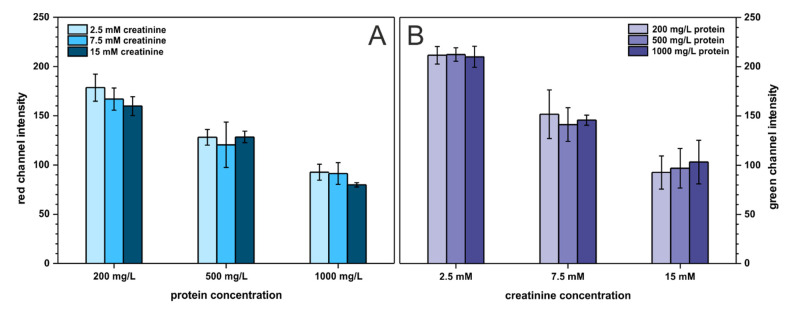
Cross-reactivity study. Bars in (**A**) represent the registered signal for protein determination with varying creatinine content, *n* = 3. Bars in (**B**) represent the registered signal for creatinine determination with varying protein content, *n* = 3.

Last but not least, the cross-reactivity of the two analytes was investigated to ensure that neither protein affects creatinine determination nor creatinine influences protein determination. Creatinine and protein were studied on three arbitrary chosen concentration levels described as: low (200 mg·L^−1^ BSA, 2.5 mmol·L^−1^ creatinine), medium (500 mg·L^−1^ BSA, 7.5 mmol·L^−1^ creatinine), and high (1000 mg·L^−1^ BSA, 15 mmol·L^−1^ creatinine), resulting in nine combinations of binary mixtures of both analytes. The outcome of this experiment is presented in Figure 6. As can be seen, the presence of protein does not affect creatinine determination and the same trend is noticeable for protein determination. Only for low protein concentration, the registered signal (red channel intensity) slightly decreases with increasing creatinine content. This is probably related to increasing solution pH with an increasing creatinine (which is an amine) concentration. This affects the color of the pH-sensitive TBPB dye. Nonetheless, the developed paper-based sensors can be employed for reliable determination of two analytes—protein and creatinine—at the same time and on a single paper strip.

### 3.4. Real Sensing Scenario

To confirm the analytical usefulness of the developed paper-based analytical devices, protein to creatinine ratio recovery was determined in artificial urine samples prepared according to the recipe given in Section 2.5. However, the initial results showed a significant overestimation of the protein concentration. This was because artificial urine has quite a high buffer capacity. Citric buffer deposited in the sensor beforehand did not have enough buffer capacity to provide the acidic conditions required for the reaction between protein and TBPB dye. There are two possible solutions to this problem: either increasing the concentration of citric buffer to increase its capacity or lowering the pH of the buffer. Both of these approaches were tested and the obtained calibration curves for BSA dissolved in artificial urine are shown in Figure S5 in ESI. The results indicate that increasing the citric buffer concentration to 0.5 mol·L^−1^ while maintaining pH 4 as well as lowering the buffer’s pH to 3 gave sensitivity similar to the curve registered in the absence of artificial urine (i.e., curve in 0.15 mmol·L^−1^ NaCl). Increasing the buffer concentration above 0.5 mol·L^−1^ resulted in a decline in linearity. Taking into account the above mentioned, 0.5 mol·L^−1^, pH 4 citric buffer was selected for further experiments.

Nine artificial urine samples with different protein to creatinine ratios were prepared and subjected to the protein and creatinine determination procedure, outlined in Section 2.3. To calculate the protein and creatinine concentrations in artificial urine samples, calibration curves were recorded using BSA and creatinine standards in sodium chloride solution. The obtained results are summarized in Table 4.

The results indicate that the developed sensors can be successfully used for protein, creatinine, and protein to creatinine ratio determination in urine samples with satisfactory precision and accuracy. The PCR recovery reached 151% in the sample containing 600 mg·L^−1^ BSA and 10 mmol·L^−1^ creatinine due to significant overestimation of the protein concentration. However, it is important to highlight that in the case of such point-of-care tests, potential false positives are much less dangerous than false negatives. This is because a false positive result would probably lead to more tests, which would eventually generate an accurate result. On the other hand, a false negative result might lead to abandonment of further diagnostic procedures and disease treatment. In conclusion, the obtained results are satisfactory for such a simple, fast, and inexpensive diagnostic test, accessible for an unskilled user.

### 3.5. Towards Calibration-Free Sensors

**Figure 7:**
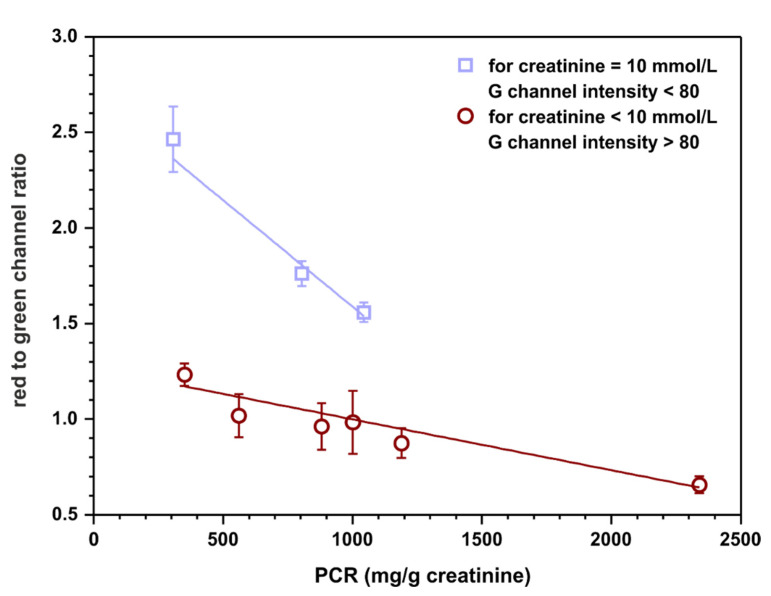
Semiquantitative, calibration-free, protein to creatinine ratio estimation. The value on ordinate represents the ratio of red channel intensity taken from the protein detection zone to green channel intensity measured in the creatinine detection zone. The top linear fit (*y* = − 0.0011 + 2.7, R^2^ = 0.930) represents samples with 10 mmol·L^−1^ of creatinine while the bottom one (*y* = − 0.0003 + 1.3, R^2^ = 0.939) represents samples with 7.5 and 4 mmol·L^−1^ of creatinine.

Calibration-free sensing devices are of significant interest as point-of-care tests. Such sensors have the potential to fulfill ‘ASSURED’ criteria (affordable, sensitive, selective, user-friendly, rapid and robust, equipment-free, deliverable to end user) developed by WHO for on-site diagnostic tests [27]. For this reason, a possibility of semiquantitative assessment of the protein to creatinine ratio based on appropriate channels intensities, without calculating the concentration of each analyte relying on previously registered calibration curves, was examined. Red channel intensities were taken from protein detection zones and green channel intensities were taken from creatinine detection zones obtained in the previous experiment (Section 3.4). The ratios of these intensities were calculated to establish if there is a correlation between this quantity and the protein to creatinine ratio. The outcome of this experiment is plotted in Figure 7.

The results indicate that there might be a possibility to fit two linear curves to the obtained data. Interestingly, one of them is for samples containing 10 mmol·L^−1^ of creatinine (and any concentration of protein, curve with squares) and the other one is for samples containing 7.5 or 4 mmol·L^−1^ of creatinine (and any concentration of protein, curve with circles). However, the assumption is that the sensors operate as calibration-free tests; therefore, the concentration of creatinine is not known a priori. As a solution, a cut-off value of green channel intensity might be introduced as an indicator of the creatinine concentration, for instance, 80. Such a procedure could potentially allow for semiquantitative protein to creatinine ratio estimation based solely on the measured RGB channel intensities. However, it is essential to highlight that a significantly bigger number of samples should be tested to obtain a more reliable fit and more certain cut-off value for the measured green channel intensity.

## 4. Conclusions

In this paper, user-friendly, inexpensive, and ecological paper-based sensors were developed for protein to creatinine ratio determination. When it comes to equipment, the analytical procedure only requires an appropriately, but rather easily and in not a complicated way, adapted smartphone. The user-friendliness and potential as a point-of-care test of this solution should be further increased by using the smartphone application for automatic signal processing, developed in an earlier study [17], provided it would be adjusted for two detection zones.

To select the method appropriate for protein determination on paper, six protein assays were tested. Only three of them, namely the bromocresol green, tetrabromophenol blue, and pyrogallol red methods, gave measurable color change and adhered to point-of-care testing principles. To the authors’ best knowledge, it is the first report on the pyrogallol red assay performed on paper. Despite excellent analytical parameters, this assay is unsuitable for long-term storage of the resulting sensors. Consequently, we recommend the tetrabromophenol blue method to be used for protein determination on paper as solid support.

Last but not least, the presented paper-based sensors for protein to creatinine ratio determination might be a step towards calibration-free sensors. However, much more research is needed combined with thorough statistical analysis to confirm the analytical usefulness of such an idea.

## Figures and Tables

**Table 1:** Protein assays chosen for comparison and the initial conditions for these methods.

Assay	Assay’s Principle	Initial Conditions	*λ*_max_	Ref.
bromocresol green (BCG)	BCG binds to albumins in acidic environment	[dye] = 0.6 mmol·L^−1^ in 0.95 mmol·L^−1^ citric buffer, pH 4.2	620 nm	[20]
pyrogallol red (PR)	PR-molybdate complex binds to proteins in acidic environment	[dye] = 0.06 mmol·L^−1^, [molybdate] = 0.04 mmol·L^−1^, in 50 mmol·L^−1^ succinic acid, 1 mmol·L^−1^ sodium oxalate, 3 mmol·L^−1^ sodium benzoate, pH 2.5	470 nm, 598 nm	[21]
ponceau S (PS)	Simultaneous PS binding and protein precipitation with TCA, pellet dissolution in sodium hydroxide	[dye] = 0.1 mmol·L^−1^ in 3% (*w*/*v*) trichloroacetic acid, 0.2 mol·L^−1^ sodium hydroxide for pellet dissolution	546 nm	[22]
Bradford	Coomassie Brilliant Blue G binds to protein in acidic environment	[dye] = 100 mg·L^−1^, dissolved in ethanol, brought to volume with 85% phosphoric acid/water 1:9 (*v*/*v*)	595 nm	[23]
BCA-Cu(II)	Cu(II) ions are reduced by peptide bonds to Cu(I) in alkaline environment, Cu(I) is detected by reaction with BCA	Reagent 1: 25 mmol·L^−1^ sodium bicinchominate, 190 mmol·L^−1^ sodium carbonate, 7 mmol·L^−1^ sodium tartate, 0.1 mol·L^−1^ sodium hydroxide, 0.1 mol·L^−1^ sodium bicarbonate, pH 11.2 Reagent 2: 0.16 mol·L^−1^ Cu(II), mix reagent 1 and reagent 2 in 50:1 (*v*/*v*) ratio	562 nm	[24]
tetrabromophenol blue (TBPB)	TBPB binds to protein in an acidic environment	[dye] = 5 mmol·L^−1^ In 250 mmol·L^−1^ citric buffer, pH 3	610 nm	[13]

**Table 2:** The results of the assays’ optimization experiments. The selected optimal parameter is highlighted in bold.

Parameter to Be Optimized	Parameter’s Value	Sensitivity [a.u./mg·L^−1^] *	R^2^	Linear Range [mg·L^−1^]
*bromocresol green method*				
dye concentration [mmol·L^−1^]	1	−0.054	0.986	0–1000
	**2.5**	**−0.057**	**0.992**	**100–1000**
	5	−0.040	0.995	100–1000
	10	−0.033	0.901	300–1000
buffer pH	3	non-linear	-	-
	4	−0.029	0.990	100–1000
	**5**	**−0.079**	**0.974**	**100–1000**
	6	−0.119	0.996	300–750
	7	non-linear	-	-
buffer concentration [mmol·L^−1^]	1	−0.072	0.990	0–1000
	**10**	**−0.080**	**0.988**	**0–1000**
	50	-0.051	0.919	0–750
	100	non-linear	-	-
ISO parameter	100	−0.062	0.983	0–1000
	**200**	**−0.078**	**0.983**	**100–1000**
	400	−0.074	0.993	100–1000
	800	−0.048	0.997	100–1000
*tetrabromophenol blue method*				
dye concentration [mmol·L^−1^]	2	−0.040	0.979	100–750
	5	−0.041	0.997	10–750
	**10**	**−0.054**	**0.966**	**100–1000**
	20	−0.045	0.993	0–750
buffer pH	2	non-linear	-	-
	3	−0.032	0.990	100–1000
	**4**	**−0.101**	**0.950**	**0–1000**
	5	−0.064	0.096	0–1000
buffer concentration [mmol·L^−1^]	10	−0.168	0.983	0–750
	100	−0.125	0.959	0–1000
	**250**	**−0.126**	**0.996**	**0–1000**
	500	−0.087	0.958	100–1000
ISO parameter	100	−0.098	0.958	0–1000
	**200**	**−0.111**	**0.991**	**0–1000**
	400	−0.055	0.998	300–1000
	800	−0.077	0.961	300–1000
*pyrogallol red method*				
dye concentration [mmol·L^−1^] **	0.05	0.0001	0.985	0–500
	0.10	0.0003	0.964	0–500
	0.25	0.0007	0.984	0–500
	**0.50**	**0.0014**	**0.999**	**0–500**
	1.00	0.0014	0.999	10–1000
molybdate concentration [mmol·L^−1^]	0.15	0.0006	0.981	0–500
	0.33	0.0010	0.991	0–500
	**0.50**	**0.0011**	**0.989**	**0–750**
	1.00	non-linear ***	-	-
buffer pH	2	non-linear	-	-
	2.5	0.0015	0.997	0–500
	**3**	**0.0014**	**0.972**	**0–750**
	4	non-linear	-	-
buffer concentration: succinate, benzoate, oxalate [mmol·L^−1^]	**10, 0.2, 0.6**	**0.0008**	**0.973**	**0–1000**
	50, 1, 3	0.0006	0.985	0–750
	100, 2, 6	0.0003	0.986	0–750
ISO parameter	**100**	**0.0008**	**0.973**	**0–1000**
	200	0.0005	0.954	0–1000
	400	0.0004	0.998	0–750
	800	0.0002	0.991	0–750

* sensitivity is expressed as [red channel intensity/mg·L^−1^] for BCG and TBPB assays and as [blue to red channel intensity/mg·L^−1^] for PR assay. ** in these experiments dye to molybdate molar ratio was kept fixed at 3:2. *** an exponential function can be fitted with the following equation: *y* = 1.45 – 0.59 exp(−0.003*x*), R^2^ = 0.997 in the range from 0 to 1000 mg·L^−1^ BSA.

**Table 3:** Analytical parameter comparison for paper-based sensors operating according to the previously optimized assays.

Analytical Parameter	Bromocresol Green	Tetrabromophenol Blue	Pyrogallol Red
LOD * [mg·L^−1^]	55	39	11
LOQ * [mg·L^−1^]	169	120	34
precision [%] (at 100 mg·L^−1^ BSA)	2.5	2.6	5.1
precision [%] (at 750 mg·L^−1^ BSA)	12	2.5	5.8

* limit of detection (LOD) and limit of quantification (LOQ) were estimated using the following equations: LOD = 3.3SD_y_/S and LOQ = 10SD_y_/S.

**Table 4:** Protein to creatinine ratio recovery study in artificial urine, *n* = 4.

Protein Concentration [mg·L^−1^]	Creatinine Concentration [mmol·L^−1^]	PCR [mg/g Creatinine]	Determined Protein Concentration [mg·L^−1^]	Determined Creatinine Concentration [mmol·L^−1^]	Determined PCR [mg/g Creatinine]	PCR Recovery [%]
300	4.0	664	302 ± 82	4.8 ± 0.8	561	85
300	7.5	354	279 ± 20	7.0 ± 0.2	350	99
300	10	265	306 ± 27	8.8 ± 0.2	307	116
600	4.0	1327	654 ± 12	4.9 ± 0.4	1188	90
600	7.5	708	584 ± 50	6.4 ± 0.3	880	114
600	10	531	846 ± 22	9.3 ± 0.2	803	151
1000	4.0	2212	918 ± 65	4.7 ± 0.4	1744	79
1000	7.5	1180	701 ± 31	6.7 ± 0.1	926	79
1000	10	885	1097 ± 130	9.3 ± 0.3	1043	118

## Supplementary Materials

The following are available online, Figure S1: Preparation of paper-based sensors for simultaneous protein and creatinine determination and measurements with the obtained sensors, Figure S2: The responses of channels from RGB color space on raising protein concentration for bromocresol green method, tetrabromophenol blue method and pyrogallol red method, Figure S3: The dependence of analytical signal on time from sample introduction for bromocresol green (BCG), tetrabromophenol blue (TBPB) methods and pyrogallol red (PR) method, Figure S4: The dependence of signal intensity on the sensor’s dipping time and time from sample introduction for protein and creatinine, Figure S5: Calibration curves registered for sensors with pre-deposited buffer solutions with varying concentrations and varying pH.

## Sample Availability

Samples of all compoundsare available from the authors.
