# Supplementary material for: Smartphone-Assisted Protein to Creatinine Ratio Determination on a Single Paper-Based Analytical Device
Source: Molecules. 2021 Oct 17;26(20):6282. doi: 10.3390/molecules26206282 (PMC8540694; doi:10.3390/molecules26206282)
Supplement: Supplementary file 1 [file molecules-26-06282-s001.zip › molecules-1386886-supplementary.pdf]

molecules

MDPI

Smartphone assisted protein to creatinine ratio determination on a single paper-based analytical device

Electronic Supplementary Information

Izabela Lewińska*, Karolina Kurdziałek and Łukasz Tymecki

University of Warsaw, Faculty of Chemistry, Pasteura 1, 02-093 Warsaw, Poland
* contact: *i.lewinska@uw.edu.pl*

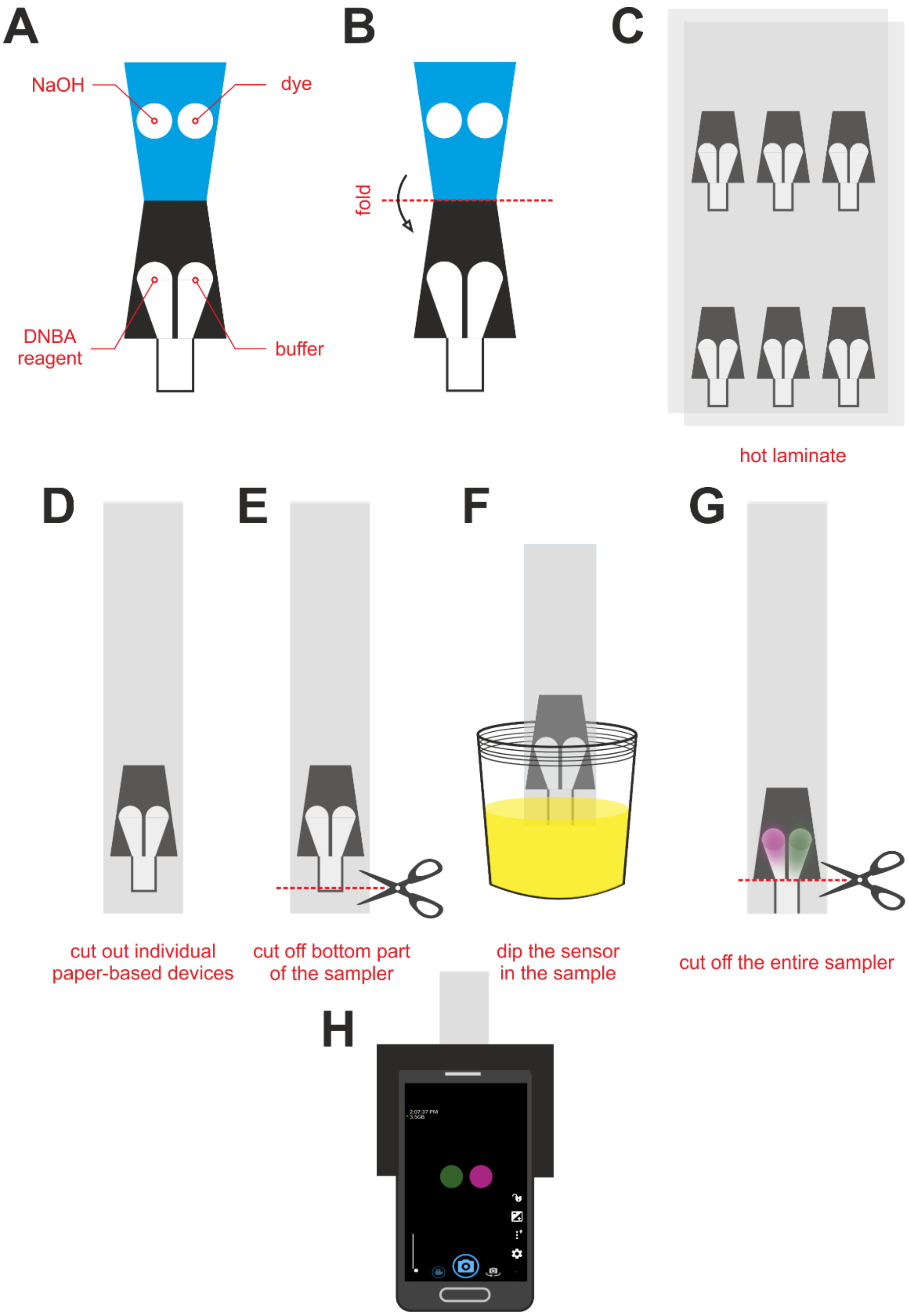

Figure S1. Preparation of paper-based sensors for simultaneous protein and creatinine determination (Steps A – D) and measurements with the obtained sensors (Steps E – H). A – drop casting reagents in the marked spots; B – after the reagents are dried, folding the sensors following the marked line; C – hot laminating the folded sensors; D – cutting out strips with individual sensors; E – cutting off the bottom part of the sampler; F – sensor's placement in the sample for a specified time; G – after the reaction has occurred, cutting off the entire sensor; H – placing the sensor in the smartphone case and acquiring a photo of the detection zones.

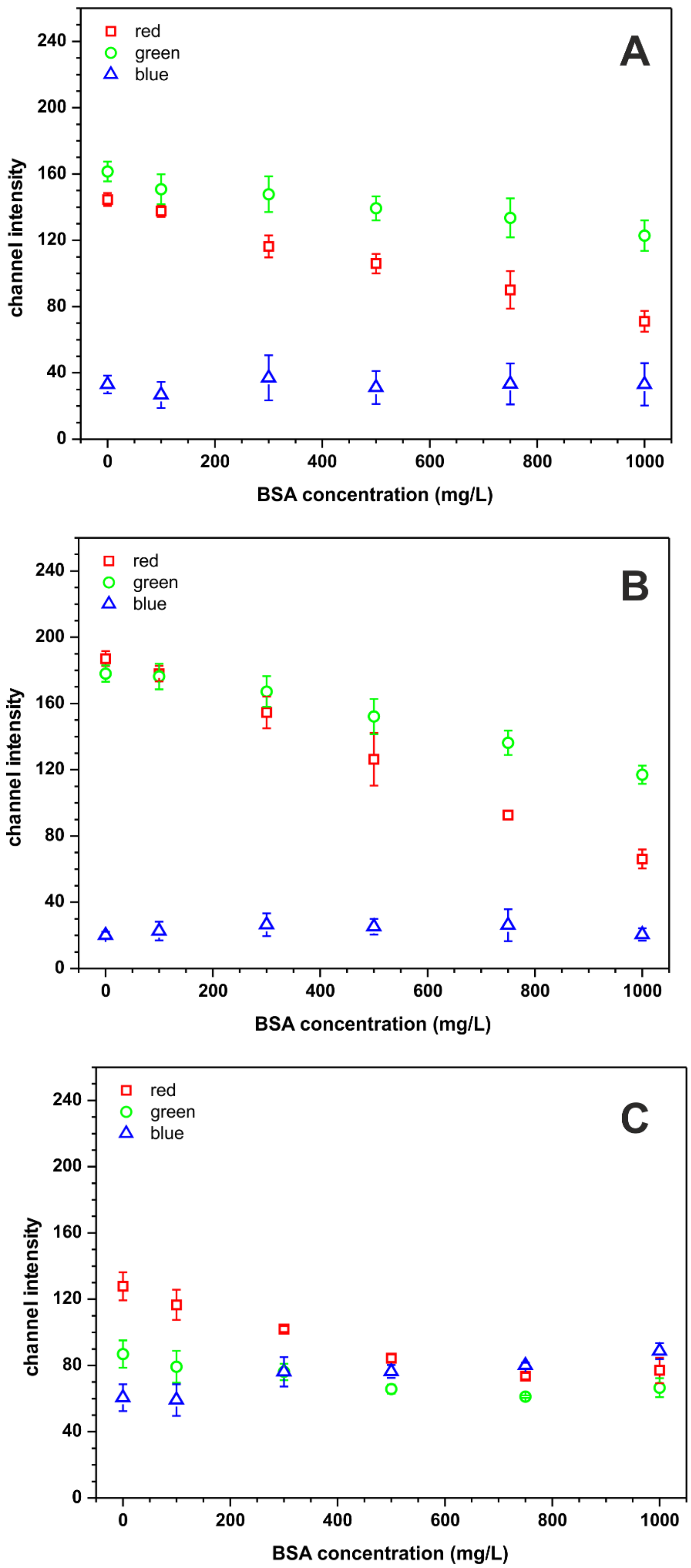

Figure S2. The responses of channels from RGB color space on raising protein concentration for (A) bromocresol green method, (B) tetrabromophenol blue method and (C) pyrogallol red method, n = 3.

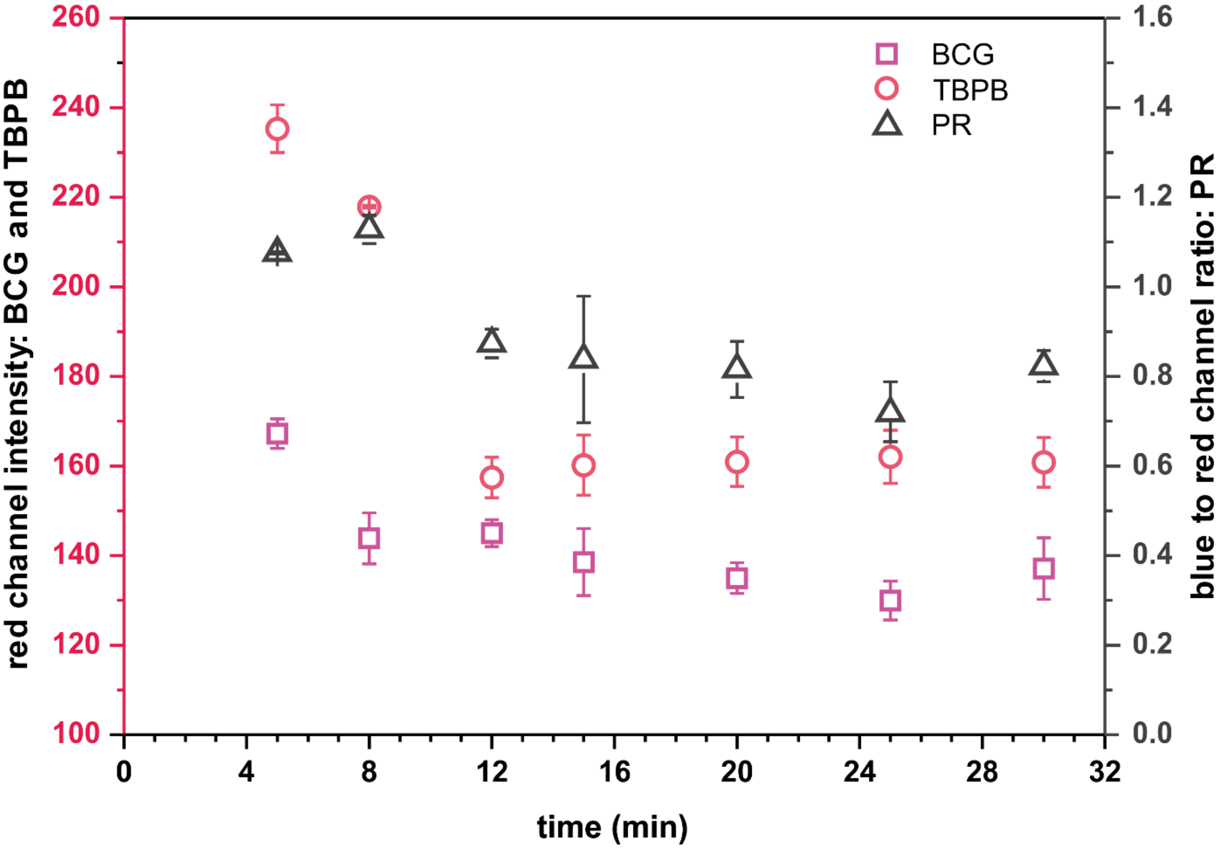

Figure S3. The dependence of analytical signal on time from sample introduction for bromocresol green (BCG), tetra-bromophenol blue (TBPB) methods – left Y-axis and for pyrogallol red (PR) method – right Y-axis. The shown kinetics were registered for 300 mg·L^{-1} of protein, n = 3.

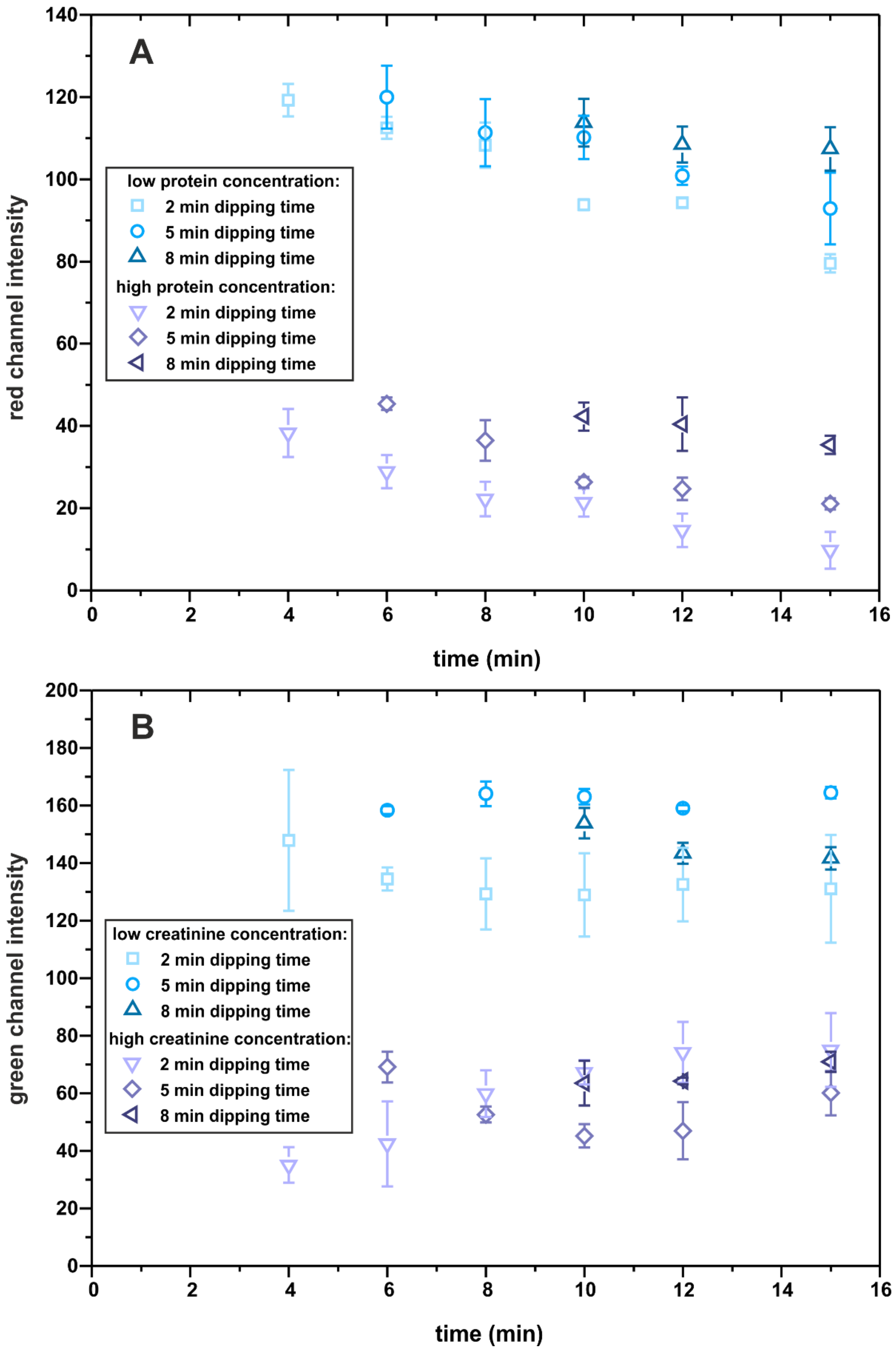

Figure S4. The dependence of signal intensity on the sensor's dipping time and time from sample introduction (total analysis time) for (A) protein (200, 1000 mg·L^{-1}) and (B) creatinine (2, 10 mmol·L^{-1}), n = 3.

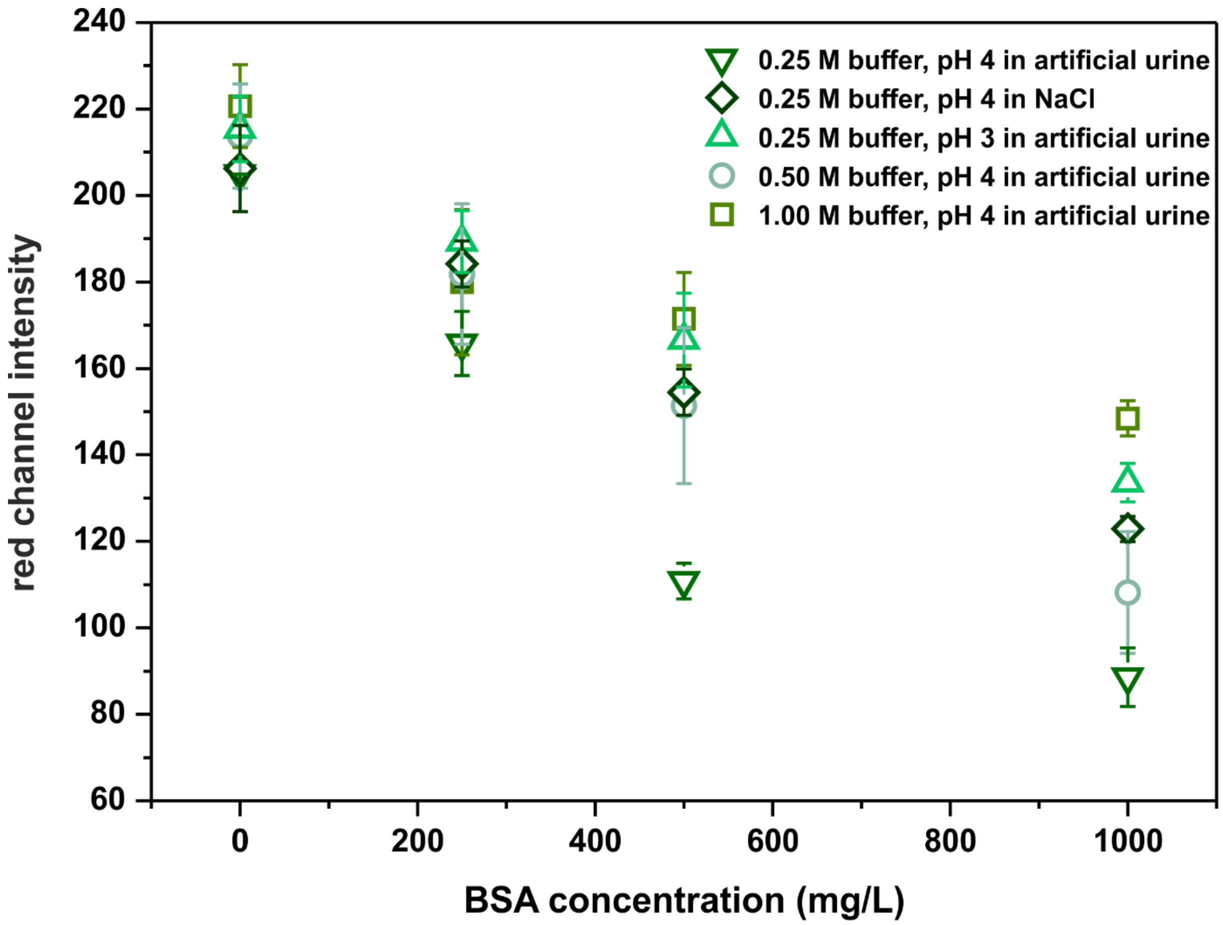

Figure S5. Calibration curves registered for sensors with pre-deposited buffer solutions with varying concentrations and varying pH, n = 3.
